# Supplementary material for: kri-1/KRIT1 promotes innate immune and lipid homeostasis in the C. elegans intestine
Source: PLoS Genet. 2026 Oct 30;22(10):e1012166. doi: 10.1371/journal.pgen.1012166 (PMC13743036; doi:10.1371/journal.pgen.1012166)
Supplement: Supplementary file 1 [file pgen.1012166.s005.docx]

**RESPONSE TO REVIEWERS**

**Editor**

*Thank you for submitting your manuscript to PLOS Genetics. After careful consideration, we feel that it has merit but does not fully meet PLOS Genetics's publication criteria as it currently stands. Therefore, we invite you to submit a revised version of the manuscript that addresses the points raised during the review process. In particular, missing information in the Methods and with regard to the GSEA datasets must be made available to the reviewers and to readers.*

We added the information requested by the reviewers to the Methods section. We added **Table S3**, which includes the datasets from the GSEA analyses. These and other changes we made to the revised manuscript are detailed in our responses below.

**Reviewer #1**

*Alaghatta and colleagues have provided a well-written and clearly presented story where a hit from a forward genetic screen with a medically relevant human ortholog is involved in innate immune responses and lipid homeostasis in C. elegans. The authors use both novel and established mutations in kri-1 to show that nonsense mutations cause induction of an irg-6 fluorescent reporter, which can be rescued with transgenic introduction of kri-1. The authors perform GSEA to show that the same genes that are upregulated during PA14 infection are up in kri-1 mutants. Accordingly, kri-1 mutants perform poorly during infection with PA14, suggesting a role for kri-1 in the innate immune response, but independent of bacterial growth/burden. They show that this innate immune response is predominantly from intestinal expression of kri-1, suggesting a role for kri-1 in the intestine. Kri-1 appears to be involved in intestinal integrity; kri-1 mutants have an increase in smurf phenotypes. To corroborate these data, the authors use an additional intestinal epithelial health marker, ACT-5, showing irregular intestinal shape and size in kri-1 RNAi animals. Kri-1 animals have decreased brood size, decreased developmental rate, but similar lifespan to wildtype animals. The authors then explore the relationship between KRI-1 and SKN-1. In their GSEA, they uncover that the set of genes differentially regulated in a skn-1 gain of function mutation has opposing regulatory patterns in kri-1 loss of function mutations. When there is reduced skn-1 expression, the irg-6 expression found in kri-1 mutants is lost. This suggests that skn-1 and kri-1 are acting in the same pathway to control the innate immune response. SKN-1 is functional in the nucleus; when kri-1 is knocked down, SKN-1 accumulates in the nucleus. This means that KRI-1 likely inhibits SKN-1 nuclear translocation and activation. Skn-1 gain of function mutants then, are expected to have similar phenotypes to kri-1 loss of function mutants. The authors observe that the skn-1 gain of function mutants are not hypersusceptible to PA14 and do not have reduced brood size. Moreover, knockdown of skn-1 cannot rescue the smurfing phenotype in kri-1 mutants. These differences are interesting and may point to more interesting biology to explore. Finally, the authors relate both of these genes to lipid metabolism. SKN-1 is known to promote germline lipids. Skn-1 gain of functions display intestinal lipid store loss. Kri-1 mutants also show this phenotype and, importantly, skn-1 downregulation rescues the kri-1-mutant-induced lipid migration. This suggests that lipid redistribution may be the mechanism through which SKN-1 and KRI-1 interact most meaningfully.

The beginning of the manuscript is very easy to follow and enjoyable to read. The title of the paper focuses on the results in Figure 4 and 5. Is there perhaps a more encompassing title?*

We changed the title (line 4): “*kri-1/KRIT1* promotes innate immune and lipid homeostasis in the *C. elegans* intestine”

*The connection between kri-1 and KRIT-1 is interesting. Could the authors highlight any gene-level similarities between these two orthologs, perhaps in figure 1B?*

We added the following text to the results section to address this point (lines 125-127): “KRI-1 is the nematode homolog of human KRIT-1, which share both 28.0% sequence identity and three core protein domains (NUDIX-5, Ankyrin repeats and a FERM B-lobe) (**Fig. 1B**).”

*Minor question: Why is the red fluorescence in Figure 1C rescue lines blown out so much?*

The presence of the *kri-1*p::*kri-1* rescue construct is verified with the *myo-3p::mCherry* co-injection marker, which stains body wall muscle of *C. elegans*. We added a sentence to the **Figure 1C** legend to clarify this point (lines 619-620).

*The data and sentences surrounding figure 4 was difficult to interpret: the use of kri-1 the gene and kri-1 the mutation was difficult to follow for a while. To improve this, first, the gain of function mutation of skn-1 should be introduced more clearly and directly. When describing the relationship between the datasets used in Figure 4A and 4B, the authors use a particularly confusing sentence pattern, despite its importance in laying the foundation for the next figure and major findings (lines 194-196). I personally do not find GSEA plots very informative, but other reviewers and readers may disagree.*

We revised the text in this section to make our explanation of these data clearer (Lines 201-209). We also more clearly introduced the *skn-1(lax188)* gain-of-function allele. The new text in this section is:

We compared previously published RNA-seq datasets of genes differentially regulated in the *kri-1(ok1251)* loss-of function with genes that are misregulated in a gain-of-function allele of *skn-*1 [*skn-1(lax188)*] [14, 29]. Genes that are activated by SKN-1 [*i.e*, upregulated in the *skn-1(lax188)* gain-of-function mutant] were significantly enriched among genes upregulated in the *kri-1(ok1251)* loss-of-function mutant (**Fig. 4A left panel, Table S3**). Conversely, genes that are suppressed during *skn-1* activation (*i.e.*, downregulated in the *skn-1(lax188)* gain-of-function mutant) were enriched among genes that were downregulated in *kri-1(ok1251)* mutants compared to wild-type controls (**Fig. 4A right panel, Table S4**). These data suggest that *kri-1* suppresses SKN-1 activation.

*Perhaps a list of the key genes that are up in kri-1 lof / down in skn-1 gof and vice versa could be useful in a supplementary table.*

We added **Table S3**, which lists these genes that drove the enrichment scores in the GSEA experiments.

*The enrichment categories are not exceptionally helpful in Figure 4B - could pathogen stress response be broken down into more sub-categories?*

The software we used to calculate gene enrichment categories does not break down pathogen-response genes into sub-categories.

*A diagram of the proposed interaction between KRI-1 and SKN-1 would be very helpful, with explanation of the cytoplasmic to nuclear transition. There is some work on HLH-30 and MML-1 that has similar dynamics.*

We included a diagram of the proposed interaction between KRI-1 and SKN-1 in **Figure 5N**.

*Is there a reason supplementary figure 1 is in supplement? It seems like it would fit quite well in Figure 5, confirming the interactions between KRI-1 and SKN-1.*

We moved the data in supplementary **Figure S1** to **Figure 5D-E**, as suggested.

*Would it be possible to do some of these assessments in a set of bonafide double mutants instead of a mutant and RNAi knockdown?*

Deletion mutants in *skn-1* are embryonic lethal, which complicates the ability to generate double mutant strains. Fortunately, *skn-1(RNAi)* is a validated tool for characterizing the role of SKN-1 in lipid mobilization from the soma to the germline [14-17]. We verified that the eggs laid by *skn-1(RNAi)* animals failed to hatch, which confirms the efficacy of *skn-1* knockdown. We added text to the Results section (Lines 247-256) to state these points. We also added this information to the Materials and Methods section (Lines 459-460).

*A summary image/diagram/figure would be most helpful at the end: explain the extent to which kri-1 and skn-1 are working in the same pathway and the ways in which they are not. The authors continuously use the word “restrains” to describe the relationship between kri-1 and skn-1, but do not explain what that means. If this is the major finding of the paper (aka the titular idea), then there can be an entire diagram explaining it. The abstract does a good job of summarizing the ideas.*

We included a diagram of the proposed interaction between KRI-1 and SKN-1 in **Figure 5N**.

*Other small questions / unintroduced ideas:
What is dod-24? Why is it important? I don’t think it is introduced at all. The other irgs are introduced briefly.*

DOD-24 is an immune response gene previously shown to be upregulated in the presence of *P. aeruginosa*. We have added context when introducing *dod-24* to make its relevance clearer (lines 137-138).

*The authors introduce the idea of toxicity of secreted phenazine toxins randomly in the explanation of figure 5. Is this important?*

We added additional context and citations to better detail the importance of phenazine toxicity in *P. aeruginosa* pathogenesis (lines 226-230).

*Methods questions:
The authors should be clear what the control is in the RNAi experiments, in the figure legends and in the methods.*

We clarified the control conditions for the RNAi experiments in the Methods section (lines 457-458) and added this information to the relevant figure legends (Lines 632-633 and 689-690).

*A description of what “intermediate” phenotypes are in the asdf and smurf phenotypes would improve understanding and interpretation. - Perhaps identifying at least one of the individual animals in the image that is in each phenotype would be useful.*

We added citations to the Materials and Methods, which provides the reference for scoring the Asdf (Lines 409-413) and Smurf (Lines 416-417) phenotypes.

*The authors state that they use a mono camera for all imaging, but provide color pictures for oil red o and smurfing images. Was a color camera used to capture these images, are these false colored, or was more image processing performed? The color images are not quantified for abundance or intensity of a color, so any approach is reasonable. Just making sure the methods are accurate.*

Color images were taken with a Zeiss Axiocam 305 color camera. We added this information to the Materials and Methods (Lines 465-466).

*Are the datasets used in the GSEA acquired by this lab or accessed from other publications? Are the results available to the readers?*

We used previously published datasets for our GSEA analyses. In the revised manuscript, we added text to more clearly state this point and better highlight the citations for these datasets (Lines 201-203).

We also added **Table S3**, which lists the genes that drove the enrichment scores in the GSEA experiments.

*Overall, this was an enjoyable read with interesting implications. The manuscript and data are solid, but could benefit from some diagrams, a bit more insight into the interaction between KRI-1 and SKN-1, and precise definition of restrain.*

We addressed these comments above.

**Reviewer #2**

*In “kri-1/KRIT1 restrains skn-1/NRF2 activation to promote innate immune and lipid homeostasis,” the authors identify KRI-1 as a factor that, when lost in the model host C. elegans, causes the constitutive activation of the immune response gene, irg-6, in a screen. It also affects other immune effectors, such as those encoded by irg-5 and dod-24. Despite the upregulation of immune response genes, loss of kri-1 results in sensitivity to the pathogen Pseudomonas aeruginosa. Specifically, this occurs when it is knocked down only in the intestine, suggesting that KRI-1 is present and plays a role in this tissue. The mammalian ortholog, KRIT1, has known roles in maintaining tissue barriers, so the effects of KRI-1 on the worm's intestinal barrier were assessed by measuring leakage of a dye through this barrier. More leakage was present following the loss of KRI-1. Additionally, disruption of the intestinal barrier was also observed using ACT-5::GFP to examine disruption of the apical actin cytoskeleton. KRI-1 was previously shown to function with SKN-1 to promote lifespan in a mechanism that depends on lipid mobilization and redistribution. Consistent with a negative regulatory effect on this transcription factor, loss of kri-1 led to the upregulation of many SKN-1-regulated genes and enhanced nuclear localization. Loss of kri-1 also resulted in age-dependent depletion of somatic fat (ASDF), like a skn-1 gain-of-function mutant. However, loss of kri-1 did not benefit pathogen survival, as mentioned – it is detrimental – and it has additional phenotypes not shared with a skn-1 gain-of-function mutant, including reduced fecundity and intestinal barrier defects. The authors thus argue that KRI-1 restrains the activity of the cytoprotective transcription factor SKN-1 to control immune gene transcription and intestinal lipid mobilization during aging, a process necessary for healthy reproduction, but that it also functions independently of SKN-1 to promote epithelial integrity and pathogen tolerance. Overall, the study is well done, and the data support the conclusions. A few suggestions to further improve the manuscript are noted.*

*1) Epistasis analysis with a skn-1 loss-of-function mutant, such as the zu135 allele, would increase the rigor of some conclusions. In particular, this applies to the lipid staining experiment in Fig. 5. The Fig. 5J-K data using skn-1 RNAi were consistent with kri-1 and skn-1 having opposite phenotypes, and skn-1 being epistatic to kri-1, but using a kri-1;skn-1double mutant would strengthen the conclusion.*

In the revised manuscript, we add text to the Results section, as follows (Lines 247-256): “To determine if *kri-1* regulates *skn-1* to control mobilization of somatic lipids, we performed *skn-1(RNAi)* in the *kri-1(ok1251)* mutant background. Of note, loss-of-function mutations in *skn-1* are embryonic lethal, which complicates the ability to generate double mutant strains in this genetic background. Fortunately, *skn-1(RNAi)* is a validated tool for characterizing the role of SKN-1 in lipid mobilization from the soma to the germline [14-17]. In this experiment, we verified that the eggs laid by *skn-1(RNAi)* animals failed to hatch, which confirms the efficacy of *skn-1* knockdown. Importantly, RNAi-mediated knockdown of *skn-1* suppressed the Asdf phenotype of *kri-1(ok1251)* mutants (**Fig. 5L-M**).”

We also added this information to our Materials and Methods (Lines 459-460).

*2) Also, using a kri-1;skn-1 double mutant in a pathogen susceptibility assay would be interesting if not perfectly conclusive. Both mutants, individually, are predicted to confer a susceptibility phenotype, and if occurring independently as predicted, an additive effect is expected.*

Please see our response to point #1 above.

*3) Fig. 5A and 5B are switched according to the text in lines 210-217.*

We corrected this mistake by switching the panels in **Fig. 5A and 5B.**

*4) line 226-227: “age-dependent depletion of somatic fat (ASDF)?” The acronym does not line up with the definition.*

We corrected the name of the acronym (lines 239-240).

**Reviewer #3**

*This paper by Alaghatta et al identifies the C. elegans gene kri-1 (homlog of human KRIT1) as a regulator of innate immunity. They futher show that it is required for tolerance to PA14 infection, fecundity, development, and the integrity of the intestinal epithelium. They find that kri-1 functions partially through suppression of transcription factor skn-1, although it’s effects on epithelial integrity and infection tolerant appear to by skn-1 independent. Overall this is a solid study and the conclusions are well supported by the data. However, there is one major issue of missing methods information that needs to be addressed as well as a few minor issues.*

We added the requested information to the methods, as detailed below.

*Major concern:
1) My major concern is that there is no description of how any of the qRT-PCR data was produced. There is no Methods section pertaining to the RNA extraction/cDNA synthesis or the qRT-PCR set up. The figure legends state that the genes of interest were normalized to a control gene, but the control gene(s) are never named. The raw data provided in Table S1 also do not include this information, and appears to show already-normalized fold changes. It is unclear from table S1 whether technical replicates were performed, and also what method was used to calculate fold changes. The qRT-PCR primers used should also be provided with the other oligos in Table S2.*

We inadvertently omitted information on the qRT-PCR experiments from our original manuscript. In this revision, we added a section to the Materials and Methods Section that includes the method of RNA extraction and cDNA synthesis, a reference to the primers that were used in this study, the names of the control genes that were used and the requested details about the method of normalization (Lines 441-447).

*Minor concerns:
1) In the Figure legend for 3E, the authors state “each data point is the average brood size from three or four animals”, but in the supplementary file provided the data for Figure 3E shows that each trial was the average of 2 animals. This also applies to Figure 5C – the legend states the data points are the average brood size from 3 animals, but table S1 shows an n of 2 for each trial. This discrepancy should be resolved.*

For **Fig. 3E**, we clarified in the figure legend that “each data point is the average brood size from four biological replicate experiments per genotype (each replicate with two animals) (Lines 654-655).”

For **Fig. 5C**, we clarified in the figure legend that “each data point is the average brood size from three biological replicate experiments per genotype (each replicate with two animals)(Lines 680-682).”

*2) For Figure 4B, the lists of genes upregulated in each condition (and their overlap) should be provided as supplementary data.*

We used previously published datasets for our GSEA analyses. In the revised manuscript, we added new text to this more clear and included citations for these datasets in the text of the paper (Lines 201-209).

We added **Table S3**, which lists the genes that drove the enrichment scores in the GSEA experiments.

Please see also our response above to a similar critique from Reviewer 1.

*3) There appears to be an error in the Results describing Figure 5 (lines 212-217). The text lists Figure 5A as the “slow kill” assay, and Figure 5B as the “fast kill” assay, but from the figure and the figure legend the text has switched them. 5A is the fast kill and 5B is the slow kill.*

We corrected this mistake by switching the panels in **Fig. 5A and 5B**.
