# Supplementary material for: kri-1/KRIT1 promotes innate immune and lipid homeostasis in the C. elegans intestine
Source: PLoS Genet. 2026 Oct 30;22(10):e1012166. doi: 10.1371/journal.pgen.1012166 (PMC13743036; doi:10.1371/journal.pgen.1012166)
Supplement: Supplementary file 2 [file pgen.1012166.s006.docx]

**RESPONSE TO REVIEWERS**

**Reviewer #2**

*Unfortunately, my major comment to the authors was not addressed. I asked about using a kri-1;skn-1 double mutant. The authors bring up the fact that skn-1 mutants are embryonic lethal, which is true, but a heterozygous animal can give rise to skn-1 homozygous mutants - they get the needed mRNA from their mothers to develop normally - and can be tested in various assays. These mutant animals will then produce dead eggs due to embryonic lethality. Therefore, the line is maintained using a balancer (unc), and the skn-1 homozygous mutants are separated out because they move normally. This description for EU31, the strain containing the zu135 allele, is given on the Caenorhabditis Genetics Center website. While difficult to cross to other strains because of these genetics, as long as the other mutation is not on the same chromosome as skn-1, it can and has been done. At the least, it would be relatively easy to expose the skn-1 mutant animals to kri-1 RNAi to validate the epistatic relationship shown in Figure 5N.*

*I am not going to insist that this be done in order for this paper to be published, but I wanted to make sure that the authors are aware that skn-1 homozygous mutants can be obtained and used in experiments – the movement phenotypes are pretty clear.*

Because *skn-1(RNAi)* is a validated tool for characterizing the role of SKN-1 in lipid mobilization from the soma to the germline [14-17], we chose not to perform this experiment.

**Reviewer #3** *This new version of the paper has edits that have significantly improved the clarity and strengthened the story, for which I commend the authors. They have satisfactorily addressed each of my minor concerns, and have added details describing the qRT-PCR experiments to the Methods to address my major concern. However, there remain two small but critical omissions that prevent the qRT-PCR methods as written from meeting the standard of containing sufficient information to allow the findings to be reproduced:

1) What method was used for the RNA extraction (Trizol phenol-chloroform? Columns?) The text states only that RNA was extracted.*

This information was added to the Materials and Methods section (line 441).

*2) Although it is helpful to clarify that the primers are from a previous publication the reference cited also does not contain the actual primer sequences, only that they are available upon request. The primer sequences should be included.*

The sequences for the qRT-PCR primers were added to Table S2, which is noted in the Materials and Methods section (lines 443-444).

*Other than these details, the paper is very strong and the conclusions well-supported.*
