# Supplementary material for: kri-1/KRIT1 promotes innate immune and lipid homeostasis in the C. elegans intestine
Source: PLoS Genet. 2026 Oct 30;22(10):e1012166. doi: 10.1371/journal.pgen.1012166 (PMC13743036; doi:10.1371/journal.pgen.1012166)
Supplement: Supplementary file 3 [file pgen.1012166.s007.docx]

**RESPONSE TO REVIEWERS**

This text was copied from the cover letter.

To address the concern regarding the use of RNAi-mediated knockdown of *skn-1,* we substantially revised the manuscript with several goals in mind: (a) We restructured the manuscript to focus on the characterization of *kri-1* – novel insights that are each substantiated by rigorous genetic experiments*;* (b) We improved our discussion of the caveats associated with the use of *skn-1(RNAi)*; (c) We softened the language that summarizes the experiments where *skn-1(RNAi)* was used. Specifically,:

1. We restructured the Results section and the presentation of data in the Figures to emphasize the characterization of *kri-1.* The new section titles of the Results are:

- Forward genetic screen identifies *kri-1*/*KRIT1* as a suppressor of innate immune gene transcription
- Innate immune dysregulation in *kri-1* mutants compromises host tolerance to infection
- *kri-1/KRIT1* supports intestinal barrier integrity
- KRI-1/KRIT1 suppresses activation of the cytoprotective transcription factor SKN-1/NRF2.
- KRI-1/KRIT1 promotes redistribution of somatic lipids from intestinal epithelial cells to the germline to ensure reproductive and evolutionary fitness

Importantly, we are pleased that the experiments conducted to support these core conclusions of our manuscript were each conducted in loss-of-function mutants of *kri-1*, often using multiple mutant alleles examined together with *kri-1* rescue strains in which this gene is expressed under the control of its own promoter in the *kri-1* loss-of-function mutant. Also of note, the restructured manuscript no longer includes the section titled “KRI-1/KRIT1 restrains SKN-1/NRF2 activity to control intestinal lipid mobilization during aging,” which relied on the experiments with *skn-1(RNAi)* to make this conclusion.

2. We improved our discussion of the caveats with the use of *skn-1(RNAi).* We added a new paragraph to the results section (lines 245-254), which I copied here:

“An important caveat of this experiment is that we were unable to confirm this observation using *skn-1* mutants. *C. elegans* *skn-1* loss-of-function mutants are embryonic lethal, which complicated our ability to generate a *kri-1* and *skn-1* double mutant strain. However, the overlap in gene expression profiles between the *skn-1(lax188)* gain-of-function mutant and *kri-1(ok1251)* loss-of-function mutants (**Fig. 4A-B**), the studies of nuclear translocation of SKN-1::GFP (**Fig. 4G-H**), and the similarities in the lipid mobilization phenotype between the *skn-1(lax188)* mutant and *kri-1(ok1251)* mutants (**Fig. 6A-B**), taken together with the observation that *skn-1(RNAi)* suppressed the lipid mobilization defect of *kri-1(ok1251)* mutants (**Fig. 6G-H**), suggests that KRI-1 restrains SKN-1/NRF2 in this context. Further studies, such as determining if KRI-1 physically binds to SKN-1, are needed to prove this interaction.”

3. We softened the language that summarizes the experiments where *skn-1(RNAi)* was used, which includes edits to the Abstract (line 29); Introduction (line 94); Results (lines 245-254); and Discussion (lines 277-278).

I also realize now that I also should have noted with our last submission the practical considerations that underlie our hesitation to perform experiments with the *skn-1* loss-of-function mutant. First, we are concerned that these experiments cannot be performed within the 30-day period required for papers at *PLOS Genetics* with Minor Revisions. Second, and most importantly, we currently lack the personnel to perform these experiments in my laboratory. This project was the dissertation research project for the first author of this manuscript, Akshay Alaghatta, who defended his PhD thesis on May 5, 2026 and subsequently moved on from my laboratory. Finally, we interpreted the comment from Reviewer 2 that conducting the experiments was not a requirement of theirs for acceptance of our manuscript (“*I am not going to insist that this be done in order for this paper to be published.” Reviewer 2).* However, I can see now that I did not do a good enough job in the prior submission of acknowledging this concern and addressing the caveats of this approach in the manuscript itself. In this revised version of this paper, I think that our manuscript is clearer and emphasizes novel conclusions that are each supported using rigorous genetic approaches. Thus, as often happens, our manuscript has been improved by the peer review process.
