# Supplementary material for: kri-1/KRIT1 promotes innate immune and lipid homeostasis in the C. elegans intestine
Source: PLoS Genet. 2026 Oct 30;22(10):e1012166. doi: 10.1371/journal.pgen.1012166 (PMC13743036; doi:10.1371/journal.pgen.1012166)
Supplement: Peer review history

# Peer review: Decision Letter 0

**DOI:** 10.1371/journal.pgen.1012166.r001

**Authors:** Paula Cohen, Cathy Savage-Dunn

9 Jul 2026

PGENETICS-D-26-00513

kri-1/KRIT1 restrains skn-1/NRF2 activation to promote innate immune and lipid homeostasis

PLOS Genetics

Dear Dr. Pukkila-Worley,

Thank you for submitting your manuscript to PLOS Genetics. After careful consideration, we feel that it has merit but does not fully meet PLOS Genetics's publication criteria as it currently stands. Therefore, we invite you to submit a revised version of the manuscript that addresses the points raised during the review process. In particular, missing information in the Methods and with regard to the GSEA datasets must be made available to the reviewers and to readers.

Please submit your revised manuscript by Sep 07 2026 11:59PM. If you will need more time than this to complete your revisions, please reply to this message or contact the journal office at plosgenetics@plos.org. When you're ready to submit your revision, log on to https://www.editorialmanager.com/pgenetics/ and select the 'Submissions Needing Revision' folder to locate your manuscript file.

Please include the following items when submitting your revised manuscript:

* A letter that responds to each point raised by the editor and reviewer(s). You should upload this letter as a separate file labeled 'Response to Reviewers'. This file does not need to include responses to any formatting updates and technical items listed in the 'Journal Requirements' section below.

* A marked-up copy of your manuscript that highlights changes made to the original version. You should upload this as a separate file labeled 'Revised Manuscript with Track Changes'.

* An unmarked version of your revised paper without tracked changes. You should upload this as a separate file labeled 'Manuscript'.

If you would like to make changes to your financial disclosure, competing interests statement, or data availability statement, please make these updates within the submission form at the time of resubmission. Guidelines for resubmitting your figure files are available below the reviewer comments at the end of this letter.

As the corresponding author, your ORCID iD is verified in the submission system and will appear in the published article. PLOS supports the use of ORCID, and we encourage all coauthors to register for an ORCID iD and use it as well. Please encourage your coauthors to verify their ORCID iD within the submission system before final acceptance, as unverified ORCID iDs will not appear in the published article. Only the individual author can complete the verification step; PLOS staff cannot verify ORCID iDs on behalf of authors.

We look forward to receiving your revised manuscript.

Kind regards,

Cathy Savage-Dunn

Academic Editor

PLOS Genetics

Paula Cohen

Section Editor

PLOS Genetics

Aimée Dudley

Editor-in-Chief

PLOS Genetics

Anne Goriely

Editor-in-Chief

PLOS Genetics

**Additional Editor Comments (if provided):**

**Journal Requirements:**

If the reviewer comments include a recommendation to cite specific previously published works, please review and evaluate these publications to determine whether they are relevant and should be cited. There is no requirement to cite these works unless the editor has indicated otherwise.

1) We ask that a manuscript source file is provided at Revision. Please upload your manuscript file as a .doc, .docx, .rtf or .tex. If you are providing a .tex file, please upload it under the item type u2018LaTeX Source Fileu2019 and leave your .pdf version as the item type u2018Manuscriptu2019.

2) Please provide an Author Summary. This should appear in your manuscript between the Abstract (if applicable) and the Introduction, and should be 150-200 words long. The aim should be to make your findings accessible to a wide audience that includes both scientists and non-scientists. Sample summaries can be found on our website under Submission Guidelines:

https://journals.plos.org/plosgenetics/s/submission-guidelines#loc-parts-of-a-submission

3) Please upload all main figures as separate Figure files in .tif or .eps format. For more information about how to convert and format your figure files please see our guidelines:

https://journals.plos.org/plosgenetics/s/figures

4)Please note that your Data Availability Statement is currently missing the DOI/accession number of each dataset OR a direct link to access each dataset. If your manuscript is accepted for publication, you will be asked to provide these details on a very short timeline. We therefore suggest that you provide this information now, though we will not hold up the peer review process if you are unable.

**Reviewers' comments:**

Reviewer's Responses to Questions

**Comments to the Authors:**

**Please note here if the review is uploaded as an attachment.**

Reviewer #1: Alaghatta and colleagues have provided a well-written and clearly presented story where a hit from a forward genetic screen with a medically relevant human ortholog is involved in innate immune responses and lipid homeostasis in C. elegans. The authors use both novel and established mutations in kri-1 to show that nonsense mutations cause induction of an irg-6 fluorescent reporter, which can be rescued with transgenic introduction of kri-1. The authors perform GSEA to show that the same genes that are upregulated during PA14 infection are up in kri-1 mutants. Accordingly, kri-1 mutants perform poorly during infection with PA14, suggesting a role for kri-1 in the innate immune response, but independent of bacterial growth/burden. They show that this innate immune response is predominantly from intestinal expression of kri-1, suggesting a role for kri-1 in the intestine. Kri-1 appears to be involved in intestinal integrity; kri-1 mutants have an increase in smurf phenotypes. To corroborate these data, the authors use an additional intestinal epithelial health marker, ACT-5, showing irregular intestinal shape and size in kri-1 RNAi animals. Kri-1 animals have decreased brood size, decreased developmental rate, but similar lifespan to wildtype animals. The authors then explore the relationship between KRI-1 and SKN-1. In their GSEA, they uncover that the set of genes differentially regulated in a skn-1 gain of function mutation has opposing regulatory patterns in kri-1 loss of function mutations. When there is reduced skn-1 expression, the irg-6 expression found in kri-1 mutants is lost. This suggests that skn-1 and kri-1 are acting in the same pathway to control the innate immune response. SKN-1 is functional in the nucleus; when kri-1 is knocked down, SKN-1 accumulates in the nucleus. This means that KRI-1 likely inhibits SKN-1 nuclear translocation and activation. Skn-1 gain of function mutants then, are expected to have similar phenotypes to kri-1 loss of function mutants. The authors observe that the skn-1 gain of function mutants are not hypersusceptible to PA14 and do not have reduced brood size. Moreover, knockdown of skn-1 cannot rescue the smurfing phenotype in kri-1 mutants. These differences are interesting and may point to more interesting biology to explore.

Finally, the authors relate both of these genes to lipid metabolism. SKN-1 is known to promote germline lipids. Skn-1 gain of functions display intestinal lipid store loss. Kri-1 mutants also show this phenotype and, importantly, skn-1 downregulation rescues the kri-1-mutant-induced lipid migration. This suggests that lipid redistribution may be the mechanism through which SKN-1 and KRI-1 interact most meaningfully.

The beginning of the manuscript is very easy to follow and enjoyable to read. The title of the paper focuses on the results in Figure 4 and 5. Is there perhaps a more encompassing title?

The connection between kri-1 and KRIT-1 is interesting. Could the authors highlight any gene-level similarities between these two orthologs, perhaps in figure 1B?

Minor question: Why is the red fluorescence in Figure 1C rescue lines blown out so much?

The data and sentences surrounding figure 4 was difficult to interpret: the use of kri-1 the gene and kri-1 the mutation was difficult to follow for a while. To improve this, first, the gain of function mutation of skn-1 should be introduced more clearly and directly.

When describing the relationship between the datasets used in Figure 4A and 4B, the authors use a particularly confusing sentence pattern, despite its importance in laying the foundation for the next figure and major findings (lines 194-196). I personally do not find GSEA plots very informative, but other reviewers and readers may disagree. Perhaps a list of the key genes that are up in kri-1 lof / down in skn-1 gof and vice versa could be useful in a supplementary table. The enrichment categories are not exceptionally helpful in Figure 4B - could pathogen stress response be broken down into more sub-categories? A diagram of the proposed interaction between KRI-1 and SKN-1 would be very helpful, with explanation of the cytoplasmic to nuclear transition. There is some work on HLH-30 and MML-1 that has similar dynamics.

Is there a reason supplementary figure 1 is in supplement? It seems like it would fit quite well in Figure 5, confirming the interactions between KRI-1 and SKN-1. Would it be possible to do some of these assessments in a set of bonafide double mutants instead of a mutant and RNAi knockdown?

A summary image/diagram/figure would be most helpful at the end: explain the extent to which kri-1 and skn-1 are working in the same pathway and the ways in which they are not. The authors continuously use the word “restrains” to describe the relationship between kri-1 and skn-1, but do not explain what that means. If this is the major finding of the paper (aka the titular idea), then there can be an entire diagram explaining it. The abstract does a good job of summarizing the ideas.

Other small questions / unintroduced ideas:

What is dod-24? Why is it important? I don’t think it is introduced at all. The other irgs are introduced briefly.

The authors introduce the idea of toxicity of secreted phenazine toxins randomly in the explanation of figure 5. Is this important?

Methods questions:

The authors should be clear what the control is in the RNAi experiments, in the figure legends and in the methods.

A description of what “intermediate” phenotypes are in the asdf and smurf phenotypes would improve understanding and interpretation.

- Perhaps identifying at least one of the individual animals in the image that is in each phenotype would be useful.

The authors state that they use a mono camera for all imaging, but provide color pictures for oil red o and smurfing images. Was a color camera used to capture these images, are these false colored, or was more image processing performed? The color images are not quantified for abundance or intensity of a color, so any approach is reasonable. Just making sure the methods are accurate.

Are the datasets used in the GSEA acquired by this lab or accessed from other publications? Are the results available to the readers?

Overall, this was an enjoyable read with interesting implications. The manuscript and data are solid, but could benefit from some diagrams, a bit more insight into the interaction between KRI-1 and SKN-1, and precise definition of restrain.

Reviewer #2: In “kri-1/KRIT1 restrains skn-1/NRF2 activation to promote innate immune and lipid homeostasis,” the authors identify KRI-1 as a factor that, when lost in the model host C. elegans, causes the constitutive activation of the immune response gene, irg-6, in a screen. It also affects other immune effectors, such as those encoded by irg-5 and dod-24. Despite the upregulation of immune response genes, loss of kri-1 results in sensitivity to the pathogen Pseudomonas aeruginosa. Specifically, this occurs when it is knocked down only in the intestine, suggesting that KRI-1 is present and plays a role in this tissue. The mammalian ortholog, KRIT1, has known roles in maintaining tissue barriers, so the effects of KRI-1 on the worm's intestinal barrier were assessed by measuring leakage of a dye through this barrier. More leakage was present following the loss of KRI-1. Additionally, disruption of the intestinal barrier was also observed using ACT-5::GFP to examine disruption of the apical actin cytoskeleton. KRI-1 was previously shown to function with SKN-1 to promote lifespan in a mechanism that depends on lipid mobilization and redistribution. Consistent with a negative regulatory effect on this transcription factor, loss of kri-1 led to the upregulation of many SKN-1-regulated genes and enhanced nuclear localization. Loss of kri-1 also resulted in age-dependent depletion of somatic fat (ASDF), like a skn-1 gain-of-function mutant. However, loss of kri-1 did not benefit pathogen survival, as mentioned – it is detrimental – and it has additional phenotypes not shared with a skn-1 gain-of-function mutant, including reduced fecundity and intestinal barrier defects. The authors thus argue that KRI-1 restrains the activity of the cytoprotective transcription factor SKN-1 to control immune gene transcription and intestinal lipid mobilization during aging, a process necessary for healthy reproduction, but that it also functions independently of SKN-1 to promote epithelial integrity and pathogen tolerance. Overall, the study is well done, and the data support the conclusions. A few suggestions to further improve the manuscript are noted.

1) Epistasis analysis with a skn-1 loss-of-function mutant, such as the zu135 allele, would increase the rigor of some conclusions. In particular, this applies to the lipid staining experiment in Fig. 5. The Fig. 5J-K data using skn-1 RNAi were consistent with kri-1 and skn-1 having opposite phenotypes, and skn-1 being epistatic to kri-1, but using a kri-1;skn-1double mutant would strengthen the conclusion.

2) Also, using a kri-1;skn-1 double mutant in a pathogen susceptibility assay would be interesting if not perfectly conclusive. Both mutants, individually, are predicted to confer a susceptibility phenotype, and if occurring independently as predicted, an additive effect is expected.

3) Fig. 5A and 5B are switched according to the text in lines 210-217.

4) line 226-227: “age-dependent depletion of somatic fat (ASDF)?” The acronym does not line up with the definition.

Reviewer #3: This paper by Alaghatta et al identifies the C. elegans gene kri-1 (homlog of human KRIT1) as a regulator of innate immunity. They futher show that it is required for tolerance to PA14 infection, fecundity, development, and the integrity of the intestinal epithelium. They find that kri-1 functions partially through suppression of transcription factor skn-1, although it’s effects on epithelial integrity and infection tolerant appear to by skn-1 independent. Overall this is a solid study and the conclusions are well supported by the data. However, there is one major issue of missing methods information that needs to be addressed as well as a few minor issues.

Major concern:

1) My major concern is that there is no description of how any of the qRT-PCR data was produced. There is no Methods section pertaining to the RNA extraction/cDNA synthesis or the qRT-PCR set up. The figure legends state that the genes of interest were normalized to a control gene, but the control gene(s) are never named. The raw data provided in Table S1 also do not include this information, and appears to show already-normalized fold changes. It is unclear from table S1 whether technical replicates were performed, and also what method was used to calculate fold changes. The qRT-PCR primers used should also be provided with the other oligos in Table S2.

Minor concerns:

1) In the Figure legend for 3E, the authors state “each data point is the average brood size from three or four animals”, but in the supplementary file provided the data for Figure 3E shows that each trial was the average of 2 animals. This also applies to Figure 5C – the legend states the data points are the average brood size from 3 animals, but table S1 shows an n of 2 for each trial. This discrepancy should be resolved.

2) For Figure 4B, the lists of genes upregulated in each condition (and their overlap) should be provided as supplementary data.

3) There appears to be an error in the Results describing Figure 5 (lines 212-217). The text lists Figure 5A as the “slow kill” assay, and Figure 5B as the “fast kill” assay, but from the figure and the figure legend the text has switched them. 5A is the fast kill and 5B is the slow kill.

**********

**Have all data underlying the figures and results presented in the manuscript been provided?**

Large-scale datasets should be made available via a public repository as described in the *PLOS Genetics*  data availability policy, and numerical data that underlies graphs or summary statistics should be provided in spreadsheet form as supporting information.

Reviewer #1: **No:**  Perhaps this is coming in a different format, but the datasets generated (or used?) to do their GSEA should be available, as well as the output of those analyses. I think there is some interesting stuff in there that should be included in a format that readers can access.

Reviewer #2: Yes

Reviewer #3: None

**********

PLOS authors have the option to publish the peer review history of their article (what does this mean?). If published, this will include your full peer review and any attached files.

If you choose “no”, your identity will remain anonymous but your review may still be made public.

**Do you want your identity to be public for this peer review?**  For information about this choice, including consent withdrawal, please see our Privacy Policy.

Reviewer #1: No

Reviewer #2: No

Reviewer #3: No

[NOTE: If reviewer comments were submitted as an attachment file, they will be attached to this email and accessible via the submission site. Please log into your account, locate the manuscript record, and check for the action link "View Attachments". If this link does not appear, there are no attachment files.]

**Figure resubmission:**

While revising your submission, we strongly recommend that you use PLOS’s NAAS tool (https://ngplosjournals.pagemajik.ai/artanalysis) to test your figure files. NAAS can convert your figure files to the TIFF file type and meet basic requirements (such as print size, resolution), or provide you with a report on issues that do not meet our requirements and that NAAS cannot fix.

After uploading your figures to PLOS’s NAAS tool - https://ngplosjournals.pagemajik.ai/artanalysis, NAAS will process the files provided and display the results in the "Uploaded Files" section of the page as the processing is complete. If the uploaded figures meet our requirements (or NAAS is able to fix the files to meet our requirements), the figure will be marked as "fixed" above. If NAAS is unable to fix the files, a red "failed" label will appear above. When NAAS has confirmed that the figure files meet our requirements, please download the file via the download option, and include these NAAS processed figure files when submitting your revised manuscript.

**Reproducibility:**

To enhance the reproducibility of your results, we recommend that authors of applicable studies deposit laboratory protocols in protocols.io, where a protocol can be assigned its own identifier (DOI) such that it can be cited independently in the future. Additionally, PLOS ONE offers an option to publish peer-reviewed clinical study protocols. Read more information on sharing protocols at https://plos.org/protocols?utm_medium=editorial-email&utm_source=authorletters&utm_campaign=protocols

# Author comment: Author response to Decision Letter 1

**DOI:** 10.1371/journal.pgen.1012166.r002

23 Jul 2026

Attachment Submitted filename: 07.15.26_ResponsetoReviewers.docx

# Peer review: Decision Letter 1

**DOI:** 10.1371/journal.pgen.1012166.r003

**Authors:** Paula Cohen, Cathy Savage-Dunn

25 Aug 2026

PGENETICS-D-26-00513R1

kri-1/KRIT1 promotes innate immune and lipid homeostasis in the C. elegans intestine

PLOS Genetics

Dear Dr. Pukkila-Worley,

Thank you for submitting your manuscript to PLOS Genetics. After careful consideration, we feel that it has merit but does not fully meet PLOS Genetics's publication criteria as it currently stands. Therefore, we invite you to submit a revised version of the manuscript that addresses the points raised during the review process. In particular, please provide the requested method details and address the comments about double mutants with skn-1.

Please submit your revised manuscript within by Sep 24 2026 11:59PM. If you will need more time than this to complete your revisions, please reply to this message or contact the journal office at plosgenetics@plos.org. When you're ready to submit your revision, log on to https://www.editorialmanager.com/pgenetics/ and select the 'Submissions Needing Revision' folder to locate your manuscript file.

Please include the following items when submitting your revised manuscript:

* A letter that responds to each point raised by the editor and reviewer(s). You should upload this letter as a separate file labeled 'Response to Reviewers'. This file does not need to include responses to formatting updates and technical items listed in the 'Journal Requirements' section below.

* A marked-up copy of your manuscript that highlights changes made to the original version. You should upload this as a separate file labeled 'Revised Manuscript with Track Changes'.

* An unmarked version of your revised paper without tracked changes. You should upload this as a separate file labeled 'Manuscript'.

If you would like to make changes to your financial disclosure, competing interests statement, or data availability statement, please make these updates within the submission form at the time of resubmission. Guidelines for resubmitting your figure files are available below the reviewer comments at the end of this letter.

As the corresponding author, your ORCID iD is verified in the submission system and will appear in the published article. PLOS supports the use of ORCID, and we encourage all coauthors to register for an ORCID iD and use it as well. Please encourage your coauthors to verify their ORCID iD within the submission system before final acceptance, as unverified ORCID iDs will not appear in the published article. Only the individual author can complete the verification step; PLOS staff cannot verify ORCID iDs on behalf of authors.

We look forward to receiving your revised manuscript.

Kind regards,

Cathy Savage-Dunn

Academic Editor

PLOS Genetics

Paula Cohen

Section Editor

PLOS Genetics

Aimée Dudley

Editor-in-Chief

PLOS Genetics

Anne Goriely

Editor-in-Chief

PLOS Genetics

**Additional Editor Comments (if provided):**

**Journal Requirements:**

If the reviewer comments include a recommendation to cite specific previously published works, please review and evaluate these publications to determine whether they are relevant and should be cited. There is no requirement to cite these works unless the editor has indicated otherwise.

1) Please amend your detailed Financial Disclosure statement. This is published with the article. It must therefore be completed in full sentences and contain the exact wording you wish to be published.

i) State what role the funders took in the study. If the funders had no role in your study, please state: "The funders had no role in study design, data collection and analysis, decision to publish, or preparation of the manuscript."

ii) If any authors received a salary from any of your funders, please state which authors and which funders..

If you did not receive any funding for this study, please simply state: u201cThe authors received no specific funding for this work.u201d

**Reviewers' comments:**

Reviewer's Responses to Questions

**Comments to the Authors:**

**Please note here if the review is uploaded as an attachment.**

Reviewer #2: Unfortunately, my major comment to the authors was not addressed. I asked about using a kri-1;skn-1 double mutant. The authors bring up the fact that skn-1 mutants are embryonic lethal, which is true, but a heterozygous animal can give rise to skn-1 homozygous mutants - they get the needed mRNA from their mothers to develop normally - and can be tested in various assays. These mutant animals will then produce dead eggs due to embryonic lethality. Therefore, the line is maintained using a balancer (unc), and the skn-1 homozygous mutants are separated out because they move normally. This description for EU31, the strain containing the zu135 allele, is given on the Caenorhabditis Genetics Center website. While difficult to cross to other strains because of these genetics, as long as the other mutation is not on the same chromosome as skn-1, it can and has been done. At the least, it would be relatively easy to expose the skn-1 mutant animals to kri-1 RNAi to validate the epistatic relationship shown in Figure 5N.

I am not going to insist that this be done in order for this paper to be published, but I wanted to make sure that the authors are aware that skn-1 homozygous mutants can be obtained and used in experiments – the movement phenotypes are pretty clear.

Reviewer #3: This new version of the paper has edits that have significantly improved the clarity and strengthened the story, for which I commend the authors. They have satisfactorily addressed each of my minor concerns, and have added details describing the qRT-PCR experiments to the Methods to address my major concern. However, there remain two small but critical omissions that prevent the qRT-PCR methods as written from meeting the standard of containing sufficient information to allow the findings to be reproduced:

1)What method was used for the RNA extraction (Trizol phenol-chloroform? Columns?) The text states only that RNA was extracted.

2)Although it is helpful to clarify that the primers are from a previous publication the reference cited also does not contain the actual primer sequences, only that they are available upon request. The primer sequences should be included.

Other than these details, the paper is very strong and the conclusions well-supported.

**********

**Have all data underlying the figures and results presented in the manuscript been provided?**

Large-scale datasets should be made available via a public repository as described in the *PLOS Genetics*  data availability policy, and numerical data that underlies graphs or summary statistics should be provided in spreadsheet form as supporting information.

Reviewer #2: Yes

Reviewer #3: Yes

**********

PLOS authors have the option to publish the peer review history of their article (what does this mean?). If published, this will include your full peer review and any attached files.

If you choose “no”, your identity will remain anonymous but your review may still be made public.

**Do you want your identity to be public for this peer review?**  For information about this choice, including consent withdrawal, please see our Privacy Policy.

Reviewer #2: No

Reviewer #3: No

[NOTE: If reviewer comments were submitted as an attachment file, they will be attached to this email and accessible via the submission site. Please log into your account, locate the manuscript record, and check for the action link "View Attachments". If this link does not appear, there are no attachment files.]

**Figure resubmission:**

While revising your submission, we strongly recommend that you use PLOS’s NAAS tool (https://ngplosjournals.pagemajik.ai/artanalysis) to test your figure files. NAAS can convert your figure files to the TIFF file type and meet basic requirements (such as print size, resolution), or provide you with a report on issues that do not meet our requirements and that NAAS cannot fix.

After uploading your figures to PLOS’s NAAS tool - https://ngplosjournals.pagemajik.ai/artanalysis, NAAS will process the files provided and display the results in the "Uploaded Files" section of the page as the processing is complete. If the uploaded figures meet our requirements (or NAAS is able to fix the files to meet our requirements), the figure will be marked as "fixed" above. If NAAS is unable to fix the files, a red "failed" label will appear above. When NAAS has confirmed that the figure files meet our requirements, please download the file via the download option, and include these NAAS processed figure files when submitting your revised manuscript.

**Reproducibility:**

To enhance the reproducibility of your results, we recommend that authors deposit laboratory protocols in protocols.io, where a protocol can be assigned its own identifier (DOI) such that it can be cited independently in the future. Additionally, PLOS ONE offers an option to publish peer-reviewed clinical study protocols. Read more information on sharing protocols at https://plos.org/protocols?utm_medium=editorial-email&utm_source=authorletters&utm_campaign=protocols

# Author comment: Author response to Decision Letter 2

**DOI:** 10.1371/journal.pgen.1012166.r004

1 Sep 2026

Attachment Submitted filename: 08.31.26_ResponsetoReviewers.docx

# Editor report: Decision Letter 2

**DOI:** 10.1371/journal.pgen.1012166.r005

**Authors:** Paula Cohen, Cathy Savage-Dunn

30 Sep 2026

PGENETICS-D-26-00513R2

kri-1/KRIT1 promotes innate immune and lipid homeostasis in the C. elegans intestine

PLOS Genetics

Dear Dr. Pukkila-Worley,

Thank you for submitting your manuscript to PLOS Genetics. After careful consideration, we feel that it has merit but does not fully meet PLOS Genetics's publication criteria as it currently stands. Therefore, we invite you to submit a revised version of the manuscript that addresses the points raised during the review process. In particular, Reviewer 2 previously raised the point that conclusions made with RNAi can be made more rigorous by analyzing mutants as well. Stating that RNAi is a well-established tool does not diminish this concern. This comment needs to be addressed in the manuscript, either with further experiments or with a discussion of the caveats.

Please submit your revised manuscript within by Oct 30 2026 11:59PM. If you will need more time than this to complete your revisions, please reply to this message or contact the journal office at plosgenetics@plos.org. When you're ready to submit your revision, log on to https://www.editorialmanager.com/pgenetics/ and select the 'Submissions Needing Revision' folder to locate your manuscript file.

Please include the following items when submitting your revised manuscript:

* A letter that responds to each point raised by the editor and reviewer(s). You should upload this letter as a separate file labeled 'Response to Reviewers'. This file does not need to include responses to formatting updates and technical items listed in the 'Journal Requirements' section below.

* A marked-up copy of your manuscript that highlights changes made to the original version. You should upload this as a separate file labeled 'Revised Manuscript with Track Changes'.

* An unmarked version of your revised paper without tracked changes. You should upload this as a separate file labeled 'Manuscript'.

If you would like to make changes to your financial disclosure, competing interests statement, or data availability statement, please make these updates within the submission form at the time of resubmission. Guidelines for resubmitting your figure files are available below the reviewer comments at the end of this letter.

As the corresponding author, your ORCID iD is verified in the submission system and will appear in the published article. PLOS supports the use of ORCID, and we encourage all coauthors to register for an ORCID iD and use it as well. Please encourage your coauthors to verify their ORCID iD within the submission system before final acceptance, as unverified ORCID iDs will not appear in the published article. Only the individual author can complete the verification step; PLOS staff cannot verify ORCID iDs on behalf of authors.

We look forward to receiving your revised manuscript.

Kind regards,

Cathy Savage-Dunn

Academic Editor

PLOS Genetics

Paula Cohen

Section Editor

PLOS Genetics

Aimée Dudley

Editor-in-Chief

PLOS Genetics

Anne Goriely

Editor-in-Chief

PLOS Genetics

**Reviewers' comments:**

[NOTE: If reviewer comments were submitted as an attachment file, they will be attached to this email and accessible via the submission site. Please log into your account, locate the manuscript record, and check for the action link "View Attachments". If this link does not appear, there are no attachment files.]

**Figure resubmission:**

While revising your submission, we strongly recommend that you use PLOS’s NAAS tool (https://ngplosjournals.pagemajik.ai/artanalysis) to test your figure files. NAAS can convert your figure files to the TIFF file type and meet basic requirements (such as print size, resolution), or provide you with a report on issues that do not meet our requirements and that NAAS cannot fix.

After uploading your figures to PLOS’s NAAS tool - https://ngplosjournals.pagemajik.ai/artanalysis, NAAS will process the files provided and display the results in the "Uploaded Files" section of the page as the processing is complete. If the uploaded figures meet our requirements (or NAAS is able to fix the files to meet our requirements), the figure will be marked as "fixed" above. If NAAS is unable to fix the files, a red "failed" label will appear above. When NAAS has confirmed that the figure files meet our requirements, please download the file via the download option, and include these NAAS processed figure files when submitting your revised manuscript.

**Reproducibility:**

To enhance the reproducibility of your results, we recommend that authors deposit laboratory protocols in protocols.io, where a protocol can be assigned its own identifier (DOI) such that it can be cited independently in the future. Additionally, PLOS ONE offers an option to publish peer-reviewed clinical study protocols. Read more information on sharing protocols at https://plos.org/protocols?utm_medium=editorial-email&utm_source=authorletters&utm_campaign=protocols

# Author comment: Author response to Decision Letter 3

**DOI:** 10.1371/journal.pgen.1012166.r006

1 Oct 2026

Attachment Submitted filename: 08.31.26_ResponsetoReviewers_auresp_3.docx

# Editor report: Decision Letter 3

**DOI:** 10.1371/journal.pgen.1012166.r007

**Authors:** Paula Cohen, Cathy Savage-Dunn

19 Oct 2026

Dear Dr Pukkila-Worley,

We are pleased to inform you that your manuscript entitled "kri-1/KRIT1 promotes innate immune and lipid homeostasis in the C. elegans intestine" has been editorially accepted for publication in PLOS Genetics. Congratulations!

Before your submission can be formally accepted and sent to production you will need to complete our formatting changes, which you will receive in a follow up email. Please be aware that it may take several days for you to receive this email; during this time no action is required by you. Please note: the accept date on your published article will reflect the date of this provisional acceptance, but your manuscript will not be scheduled for publication until the required changes have been made.

Once your paper is formally accepted, an uncorrected proof of your manuscript will be published online ahead of the final version, unless you’ve already opted out via the online submission form. If, for any reason, you do not want an earlier version of your manuscript published online or are unsure if you have already indicated as such, please let the journal staff know immediately at plosgenetics@plos.org.

In the meantime, please log into Editorial Manager at https://www.editorialmanager.com/pgenetics/, click the "Update My Information" link at the top of the page, and update your user information to ensure an efficient production and billing process. Note that PLOS requires an ORCID iD for all corresponding authors. Therefore, please ensure that you have an ORCID iD and that it is validated in Editorial Manager. To do this, go to ‘Update my Information’ (in the upper left-hand corner of the main menu), and click on the Fetch/Validate link next to the ORCID field.  This will take you to the ORCID site and allow you to create a new iD or authenticate a pre-existing iD in Editorial Manager.

If you have a press-related query, or would like to know about making your underlying data available (as you will be aware, this is required for publication), please see the end of this email. If your institution or institutions have a press office, please notify them about your upcoming article at this point, to enable them to help maximise its impact. Inform journal staff as soon as possible if you are preparing a press release for your article and need a publication date.

Thank you again for supporting open-access publishing; we are looking forward to publishing your work in PLOS Genetics!

Yours sincerely,

Cathy Savage-Dunn

Academic Editor

PLOS Genetics

Paula Cohen

Section Editor

PLOS Genetics

Aimée Dudley

Editor-in-Chief

PLOS Genetics

Anne Goriely

Editor-in-Chief

PLOS Genetics

www.plosgenetics.org

BlueSky: @plos.bsky.social

----------------------------------------------------

Comments from the reviewers (if applicable):

----------------------------------------------------

**Data Deposition**

If you have submitted a Research Article or Front Matter that has associated data that are not suitable for deposition in a subject-specific public repository (such as GenBank or ArrayExpress), one way to make that data available is to deposit it in the Dryad Digital Repository. As you may recall, we ask all authors to agree to make data available; this is one way to achieve that. A full list of recommended repositories can be found on our website.

The following link will take you to the Dryad record for your article, so you won't have to re‐enter its bibliographic information, and can upload your files directly:

http://datadryad.org/submit?journalID=pgenetics&manu=PGENETICS-D-26-00513R3

More information about depositing data in Dryad is available at http://www.datadryad.org/depositing. If you experience any difficulties in submitting your data, please contact help@datadryad.org for support.

Additionally, please be aware that our data availability policy requires that all numerical data underlying display items are included with the submission, and you will need to provide this before we can formally accept your manuscript, if not already present.

----------------------------------------------------

**Press Queries**

If you or your institution will be preparing press materials for this manuscript, or if you need to know your paper's publication date for media purposes, please inform the journal staff as soon as possible so that your submission can be scheduled accordingly. Your manuscript will remain under a strict press embargo until the publication date and time. This means an early version of your manuscript will not be published ahead of your final version. PLOS Genetics may also choose to issue a press release for your article. If there's anything the journal should know or you'd like more information, please get in touch via plosgenetics@plos.org.

# Editor report: Acceptance letter

**DOI:** 10.1371/journal.pgen.1012166.r008

**Authors:** Paula Cohen, Cathy Savage-Dunn

PGENETICS-D-26-00513R3

kri-1/KRIT1 promotes innate immune and lipid homeostasis in the C. elegans intestine

Dear Dr Pukkila-Worley,

We are pleased to inform you that your manuscript entitled "kri-1/KRIT1 promotes innate immune and lipid homeostasis in the C. elegans intestine" has been formally accepted for publication in PLOS Genetics! Your manuscript is now with our production department and you will be notified of the publication date in due course.

The corresponding author will soon be receiving a typeset proof for review, to ensure errors have not been introduced during production. Please review the PDF proof of your manuscript carefully, as this is the last chance to correct any errors. Please note that major changes, or those which affect the scientific understanding of the work, will likely cause delays to the publication date of your manuscript.

Soon after your final files are uploaded, unless you have opted out or your manuscript is a front-matter piece, the early version of your manuscript will be published online. The date of the early version will be your article's publication date. The final article will be published to the same URL, and all versions of the paper will be accessible to readers.

For Research Articles, you will receive an invoice from PLOS for your publication fee after your manuscript has reached the completed accept phase. If you receive an email requesting payment before acceptance or for any other service, this may be a phishing scheme. Learn how to identify phishing emails and protect your accounts at https://explore.plos.org/phishing.

Thank you again for supporting PLOS Genetics and open-access publishing. We are looking forward to publishing your work!

With kind regards,

Janani Seenivasan

PLOS Genetics

On behalf of:

The PLOS Genetics Team

Carlyle House, Carlyle Road, Cambridge CB4 3DN | United Kingdom

plosgenetics@plos.org | +44 (0) 1223-442823

plosgenetics.org | Twitter: @PLOSGenetics
